# Development and Validation of the Overall Foot Health Questionnaire for Patients with Rheumatoid Arthritis: A Cross-Sectional Descriptive Analysis

**DOI:** 10.3390/medicina55060290

**Published:** 2019-06-19

**Authors:** María Reina-Bueno, José Rafael González-López, Daniel López-López, César Calvo-Lobo, Marina Ballesteros-Mora, Inmaculada Rodríguez-Moreno, Pedro V. Munuera-Martínez

**Affiliations:** 1 Department of Podiatry, Faculty of Nursing, Physiotherapy and Podiatry, University of Sevilla, 41009 Sevilla, Spain; mreina1@us.es (M.R.-B.); marina_ballesteros@hotmail.com (M.B.-M.); inma10_moreno@hotmail.com (I.R.-M.); pmunuera@us.es (P.V.M.-M.); 2 Departament of Nursing, Faculty of Nursing, Physiotherapy and Podiatry, University of Sevilla, 41009 Sevilla, Spain; joserafael@us.es; 3 Research, Health and Podiatry Unit, Department of Health Sciences, Faculty of Nursing and Podiatry, University of A Coruña, 15403 Ferrol, Spain; 4 Nursing and Physical Therapy Department, Faculty of Health Sciences, University of León, 24401 Ponferrada, Spain; ccall@unileon.es

**Keywords:** arthritis, rheumatoid, Delphi technique, health education

## Abstract

*Background and Objectives*: In general, patients with rheumatoid arthritis (RA) are ignorant of the physician’s role and of RA-related feet problems. The aim of our study was to validate a questionnaire on the knowledge of different aspects of overall foot health in patients with RA. *Materials and Methods*: A cross-sectional descriptive analysis was carried out between March 2017 and April 2017. A questionnaire was designed and validated through the Delphi method to evaluate the knowledge about the illness, the repercussions on feet, medical podiatry care, and the role of the medical podiatrist. *Results*: After being checked by a panel of experts, all the items obtained a Cronbach’s alpha over 0.70. *Conclusions*: The content of this questionnaire about the knowledge of different aspects of medical podiatry health in patients with RA has internal consistency.

## 1. Introduction

Rheumatoid arthritis (RA) is a chronic inflammatory illness of the joints with a prevalence of between 0.3% and 1.5% of the total world population [1,2].

Of all patients with RA, 85% to 100% experience RA-related issues with their feet. The progression of this RA-related issues is related to the length and severity of the illness [1].

The most frequent deformities in these patients are rearfoot valgus, Hallux Abductus Valgus, and other deformities in the smaller toes [2], increasing plantar pressure and causing musculoskeletal pain [1,2,3,4].

RA can lead to a loss of function, a reduction of mobility, and a negative impact on quality of life [5]. The recommendations in both clinical practice guides and standard care, support the need of early podiatric intervention. Research has shown that it would be appropriate to include a podiatrist in the multidisciplinary team for patients with RA [5,6]. Therapeutic education is considered a key part of podiatrist-related treatment for patients with RA [7].

Therapeutic education plays a fundamental role in all chronic illnesses. The aim of the patients is to be able to improve their life [8]. There is evidence that early intervention for both potential or existing foot problems can improve the long-term results [9]. In general, patients with RA are ignorant of the podiatrist’s role and of RA-related feet problems [6].

There is currently a limited amount of research that describes the results of overall foot educational interventions in these patients [10]. Due to this need, we have proposed carrying out sessions of podiatric therapeutic education in patients with RA. These sessions consist of a health education seminar, followed by a talk. To carry out this educational intervention, and to be able to value its effectiveness, it was necessary to elaborate and validate a questionnaire that covered these aspects. Due to the limited literature on the subject [10], there is currently no validated questionnaire. For this reason, the main aim of this work was to validate a questionnaire, through the Delphi method, to increase our knowledge of different aspects of overall foot health in patients with RA.

## 2. Methods

A questionnaire was designed and validated through the Delphi method [11,12] to evaluate the patients’ knowledge about RA, the repercussions on their feet, overall foot care, and the role of nursing in their care.

This Delphi method has been widely demonstrated to be useful in achieving consensus in an area where there is a lack of empirical evidence [13]. This method is an efficient and systematic procedure that aims to compile expert opinions about a particular topic. Furthermore, a questionnaire enables a deeper understanding of these opinions [14].

The Delphi method is classified as one of the general prospective methods that seeks to achieve a better consensus of an expert group based on the analysis of and reflection on a concrete problem [13]. It is used to determine the validity of the content of an instrument; the degree to which a test appropriately represents what has been carried out [15,16]. This type of validation has been widely used in the scientific literature to validate questionnaires in different areas [11,12,14,15,16,17,18,19,20,21,22].

The study was carried out between March 2017 and April 2017. An Ethics Committee approval was obtained from the Junta de Andalucía, Spain (fe524a8b7e8159db98f0645027de03e6451e3595, application data 12 October 2016.) Two groups took part in the study: A coordinating group and an expert panel. The coordinating group was made up of two podiatrists and a professional nurse whose work takes place at the University of Seville. All of them have a Ph.D. The coordinating group worked on the design of the initial questionnaire, with a total of nine items with closed answers (Appendix A). The objective of this questionnaire was to evaluate the knowledge of these patients about four fundamental aspects related to the overall health of their feet: The repercussions of RA on their feet, the use of appropriate footwear, the importance of self-care, and the skills of nursing.

The 16 experts included in the panel answered questions of affiliation, as well as their degree of knowledge concerning RA. The questionnaire was sent by e-mail to evaluate each question on a five-point Likert scale. The contributions of each of the items were collated.

Two phases were carried out in the validation process: The preliminary phase and the exploratory phase. In the preliminary phase, the coordinator group was in charge of studying and approving the work protocol, as well as studying and ratifying the list of experts taking part. Having done this stage, the exploratory stage began in the coordinator group with four sub-stages: First, questionnaires were sent out (Likert-type scale) with a letter of presentation; second, the answers were analyzed, the following questionnaires were prepared, and the appropriate feedback was delivered; third, the results were interpreted; and fourth, the research was correctly supervised, with corrective measures taken when necessary. In a parallel manner to these sub-stages, the expert panel was in charge of answering the questionnaires [11,12].

The exploratory phase was developed through two rounds. The first round where, after analyzing the answers of the expert panel, some modifications were carried out according to its contributions, and a second round that collated the contributions in each of the items of the questionnaire, also carrying out some modifications and adding two new questions about nail cutting and treating corns.

The computer program used in the statistical analysis was SPSS version 22.0. For the study of the quantitative variables, age and years of experience, we used the average and the standard deviation. For the qualitative variables (degree, current job post, professional practice, and teaching category), we calculated the frequencies and percentages.

In each of the rounds we carried out the Cronbach alpha statistical test for each of the items and the questionnaire in general. The method of internal consistency based on Cronbach’s alpha enables the estimation of the reliability of a measurement instrument through a set of items, which it is expected will measure the same construct or theoretical dimension. The closer the alpha value is to one, the greater the internal consistency of the items analyzed is. Polit and Hungler [23], like Burns and Grove [24], consider that a reliability coefficient over a minimum value of 0.70 is in general acceptable.

## 3. Results

The coordinator group was set up as explained in the previous section. In the preliminary phase this coordinator group selected the expert panel to validate the questionnaires.

The expert panel comprised of 62.5% podiatrists, 18.8% nurses, and 18.8% who belonged to other health professions. Regarding their current jobs, 87.5% were teachers and 12.5% carried out their professional abilities in the private care area. In the following table we describe the teaching category of the members of the expert panel (Table 1). In total, 75% had PhDs, 12.5% had a university degree, and 12.5% had a masters. The degree of knowledge of the experts concerning RA on a scale of 1 to 10 was on average 6.9 ± 1.7 (range of 3 to 10). The average years of experience was 19.6 ± 5.8 years (range of 13 to 31 years).

As was described in the previous section, the initial questionnaire was designed and sent to the experts for their validation (Appendix A).

In the second part, we calculated the mean value and the standard deviation, and found the frequencies of all the different answers for each of the items. It should be highlighted that all the items obtained the qualification of very appropriate or quite appropriate from more than 70% of the experts. Items 1 and 2 attained the highest values, obtaining these qualifications from more than 90% of the experts. The Cronbach alpha statistical test was carried out for each of the items individually and for the set of them. The values were over 0.70 in all the cases, the total for the questionnaire being 0.855 (Table 2).

We analyzed the contributions of the expert panel. Some formal aspects of the different items were modified and a new item on nail cutting and the removal of corns was added. The questionnaires with these modifications were sent back to the expert panel to be valued anew (Appendix B).

In the third part, we again worked out the frequency of the answers of the expert panel regarding the different items. All of them attained a qualification of very appropriate or quite appropriate from more than 75% of the experts, attaining 100% in these qualifications for items 1, 2, and 10. Cronbach’s alpha was done again. The values were over 0.70 in all the cases, the total for the questionnaire being 0.790 (Table 3).

The contributions were analyzed anew and we modified the writing of items 9 and 10. We added a new, open-answer item in which we asked what the patients did when their feet hurt. In the last phase we had the result of the final questionnaire, which would be used later for the investigation (Appendix C).

Finally, to validate the content of this questionnaire, a pilot test was done to calculate the validity and reliability. Eleven subjects participated in this study. Cronbach’s alpha was done, the total for the questionnaire being 0.792.

## 4. Discussion

Various studies have been done that show the positive effect of therapeutic education on patients with RA about their knowledge of the illness [25], their general state [26], the degree of pain [26], the self-handling of the illness [26,27,28], the level of control of the illness, the patient’s activity [26], the physical function [29], their perception of general health [30], and satisfaction [27]. Therapeutic education plays a fundamental role in adhering to treatments in chronic illnesses and therefore increases the quality of life of these patients [31].

In 2008, Riemsma et al. [32] published a systematic review about the effects of therapeutic education in patients with RA. This type of intervention produces beneficial effects in the short term on disability, joint counts, the patient’s global evaluation, his/her psychological status, and depression. We believe that the lack of evidence of the benefits of the therapeutic treatment of patients with RA is due to there being few studies of the matter.

Foot problems are one of the most frequent in patients with RA [1,2,33]. Therapeutic education provides the appropriate information concerning the role of the podiatrist and the RA-related foot problems. It is an important tool to improve these patients’ quality of life. Various works about the opinions of patients with RA and podiatrists reflect the need for podiatric education in this group [10,34]. Graham et al. [10] carried out a literature review about podiatric therapeutic education for patients with RA. They determined that there were no specific investigations regarding the development and effects of therapeutic education in patients with RA. According to the results of these authors, there is an evident need to carry out podiatric education activities.

In 2010, Juarez et al. [34] evaluated the prevalence of foot health problems of patients with inflammatory arthritis—68% had foot problems. Only 21% were informed regarding the consequences of illness-related foot problems and general health, and 9% about footwear. The data of this study ratify the need to carry out activities about education for podiatric health.

In general, there is a lack of formal overall foot education [6]. This is why we had proposed carrying out podiatric therapeutic education sessions in patients with RA.

In our study, the average, the standard deviation, and Cronbach’s alpha was calculated for each of the items. In the Cronbach alpha statistical test values over 0.7 were obtained in all the items and as an average value of the questionnaire. All of the items attained a qualification of very appropriate or quite appropriate in more than 75% of the items, even achieving values of 100% in these categories for items 1, 2, and 10. It was considered that the items were valid if the degree of appropriateness was over 70%.

After validating the content of this questionnaire, a pilot test was done to calculate the validity and reliability. Foot Health Promotion activities will be carried out later.

Among the limitations of this work was that the expert panel might have preconceived ideas concerning the group of people with RA.

## 5. Conclusions

We conclude by stating that the content of this questionnaire worked out with the knowledge of different aspects of foot health in patients with RA had internal consistency and could be a useful tool to analyze the knowledge of distinct aspects of podiatric health in patients.

## Figures and Tables

**Table 1:** Description of the expert panel according to the teaching category of its members.

Teaching Category	Frequencies
University Full Professor	7.1%
College Full Professor	7.1%
Tenured Professor	14.3%
Contracted Professor	57.1%
Collaborator	14.3%

**Table 2:** Average, median, typical deviation, and values of the statistical Cronbach alpha test for the first round.

	Average	Median	Typical Deviation	Cronbach’s Alpha
Item 1	1.2	1	0.561	0.839
Item 2	1.1	1	0.258	0.843
Item 3	1.9	2	0.990	0.881
Item 4	1.9	1	1.246	0.845
Item 5	1.7	1	0.816	0.805
Item 6	1.3	1	0.617	0.810
Item 7	1.5	1	0.743	0.827
Item 8	1.4	1	0.737	0.842
Item 9	1.1	1	0.352	0.847

**Table 3:** Average, median, typical deviation, and values of the statistical Cronbach alpha test for the second round.

	Average	Median	Typical Deviation	Cronbach’s Alpha
Item 1	1.2	1	0.403	0.805
Item 2	1.2	1	0.414	0.803
Item 3	1.7	1	0.873	0.789
Item 4	1.3	1	0.602	0.805
Item 5	2.1	2	1.181	0.756
Item 6	1.8	1.5	1.109	0.759
Item 7	1.8	1	1.167	0.775
Item 8	1.7	1	1.291	0.790
Item 9	1.4	1	0.814	0.798
Item 10	1.3	1	0.447	0.790

## Appendix A. Questionnaire Sent to the Experts (1st. Round)

**VALIDATION WITH DELPHI METHOD OF QUESTIONNAIRE FOR PATIENTS WITH RHEUMATOID ARTHRITIS**
Dear colleague,
We are carrying out a validation of a questionnaire aimed at patients with Rheumatoid Arthritis (RA).
Through it we wish to assess the knowledge of these patients about four aspects fundamentally related with the health of their feet: Their awareness of the repercussions of RA on feet, their knowledge about the use of appropriate footwear, their awareness of the importance of self-care of their feet, and their knowledge about the skills of nursing.
To validate this questionnaire your opinion is fundamental. This is why we request that you spend a few minutes answering these questions.

**1. Personal data of the expert.**

**Full name:**
**Qualifications:**
**Current job position and company:**
**Professional qualifications:**
□ University degree □ Master □ Ph.D.
**Years of experience in the profession: ____ years.**
**Teaching category (where appropriate):**
□ University Full Professor □ Contracted doctor
□ Tenured Professor/ College □ Assistant doctor
□ Full Professor/ College □ Assistant
□ Associate Professor □ Collaborator
□ Part-time Professor □ Other

 2. Mark with a cross (X) in the box that corresponds to the degree of knowledge which you have about the research topic that we are developing, valuing on a scale from a 0 a 10 (considering 0 as not having any knowledge at all and 10 total knowledge of the subject dealt with).

**0**	**1**	**2**	**3**	**4**	**5**	**6**	**7**	**8**	**9**	**10**
										

 3. Next, we ask if you agree with the items which make up the questionnaire presented afterward. Take into account that this questionnaire is an instrument to find out and to analyze the knowledge that patients with rheumatoid arthritis have of podiatric problems related with this illness, of the appropriate footwear, of the daily care of their feet and the figure of the podiatrist. To do so, mark with a cross (X) in the column you consider suitable for each of the items.

**Items of the Questionnaire**			**Very Appropriate**	**Quite Appropriate**	**Appropriate**	**Not very Appropriate**	**Not Appropriate**
**Awareness of the repercussions of Rheumatoid Arthritis on feet**							
1. Do you believe that Rheumatoid Arthritis produces pain in feet as well as in other parts of the body?							
□ Yes	□ No	□ DK/DA					
2. Do you believe that Rheumatoid Arthritis produces deformities in feet such as: claw toes, bunions, corns, etc.?							
□ Yes	□ No	□ DK/DA					
**Knowledge about the use of appropriate footwear**							
3. Do you think that the regular use of sports footwear would be beneficial to the health of your feet?							
□ Yes	□ No	□ DK/DA					
4. Would daily use of broad footwear with a soft sole, laces and soft heel be beneficial?							
□ Yes	□ No	□ DK/DA					
**Awareness of the importance of self-care of feet**							
5. Do you believe that moisturizing cream should be applied daily?							
□ Yes	□ No	□ DK/DA					
6. Is it appropriate to dry between your toes after your daily bath?							
□ Yes	□ No	□ DK/DA					
7. How should you apply moisturizing cream?							
□ Between your toes							
□ On the back and the sole of your feet							
□ Between your toes, on the back and sole of your feet							
**Items of the Questionnaire**			**Very Appropriate**	**Quite Appropriate**	**Appropriate**	**Not very Appropriate**	**Inappropriate**
**Knowledge about the skills of the podiatrist**							
8. Do you believe that a podiatrist could improve your quality of life?							
□ Yes	□ No	□ DK/DA					
9. What’s the podiatrist’s role?							
□ Nail cutting							
□ Getting rid of calluses and corns							
□ Foot surgery							
□ Study of footsteps							
□ Making insoles							
□ Prescribing medicine							
□ X-rays							
□ Treating foot wounds							
NOTE: If you wish to make an observation about any of the items proposed or to propose a new one, you can do so here:							

“Your help is essential to achieve the aim of this study, which is why we thank you enormously for your collaboration”.

## Appendix B. Questionnaire Sent to the Experts (2nd. round)

**VALIDATION WITH DELPHI METHOD OF QUESTIONNAIRE FOR PATIENTS WITH RHEUMATOID ARTHRITIS**
Dear colleague,
We are carrying out a validation of a questionnaire aimed at patients with Rheumatoid Arthritis (RA).
Through it we wish to assess the knowledge of these patients about four aspects fundamentally related with the health of their feet: Their awareness of the repercussions of RA on feet, their knowledge about the use of appropriate footwear, their awareness about the importance of self-care of their feet, and their knowledge about the skills of nursing.
To validate this questionnaire your opinion is fundamental. This is why we request that you spend a few minutes answering these questions.

**1. Personal data of the expert.**

**Full name:**
**Qualifications:**
**Current job position and company:**
**Professional qualifications:**
□ University degree □ Master □ Ph.D.
**Years of experience in the profession: ____ years.**
**Teaching category (where appropriate):**
□ University Full Professor   □ Contracted doctor
□ Tenured Professor/ College   □ Assistant doctor
□ Full Professor/ College   □ Assistant
□ Associate Professor   □ Collaborator
□ Part-time Professor   □ Other

 2. Mark with a cross (X) in the box that corresponds to the degree of knowledge which you have about the research topic that we are developing, valuing on a scale from a 0 a 10 (considering 0 as not having any knowledge at all and 10 total knowledge of the subject dealt with).

**0**	**1**	**2**	**3**	**4**	**5**	**6**	**7**	**8**	**9**	**10**
										

 3. Next we ask your opinion regarding if you agree with the items which make up the questionnaire presented afterward. Take into account that this questionnaire is an instrument to find out and to analyze the knowledge that patients with rheumatoid arthritis have of podiatric problems related with this illness, of the appropriate footwear, of the daily care of their feet and the figure of the podiatrist. To do so, mark with a cross (X) in the column you consider suitable for each of the items.

**Questionnaire Select an Option**				**Very Appropriate**	**Quite Appropriate**	**Appropriate**	**Not very Appropriate**	**Inappropriate**
**Awareness of the repercussions of Rheumatoid Arthritis on feet**								
1. Do you believe that Rheumatoid Arthritis produces pain in feet as well as in other parts of the body?								
□ Yes	□ No	□ Does not know	□ Does not answer					
2. Rheumatoid Arthritis produces deformities in feet such as: claw toes, bunions, corns, etc.?								
□ Yes	□ No	□ Does not know	□ Does not answer					
3. Rheumatoid Arthritis can produce falls and cause difficulty in walking etc.?								
□ Yes	□ No	□ Does not know	□ Does not answer					
**Knowledge about the use of appropriate footwear**								
4. Would it be beneficial for the health of your feet to regularly use quality standard sports shoes?								
□ Yes	□ No	□ Does not know	□ Does not answer					
5. Would daily use of wide footwear, with a soft sole, with a fastening system (laces, Velcro, zips, etc.) and a low heel be beneficial?								
□ Yes	□ No	□ Does not know	□ Does not answer					
**Awareness of the importance of self-care of feet**								
6. Should you apply moisturizing cream daily?								
□ Yes	□ No	□ Does not know	□ Does not answer					
7. Is it appropriate to dry between your toes after your daily bath?								
□ Yes	□ No	□ Does not know	□ Does not answer					
8. What is the best way of cutting your toenails?								
□ Straight, trimming the tips.								
□ Straight, without trimming the tips.								
□ In a curve, trimming the tips.								
□ Does not know		□ Does not answer						
9. Is it appropriate to treat calluses and corns yourself?								
□ Yes	□ No	□ Does not know	□ Does not answer					
**Knowledge about the podiatrist’s skills**								
10. Could the intervention of a podiatrist improve your general wellbeing by acting on your feet?								
□ Yes	□ No	□ Does not know	□ Does not answer					
11. What’s the podiatrist’s role?								
□ Nail cutting.								
□ Getting rid of calluses and corns.								
□ Foot surgery.								
□ Study of footsteps.								
□ Making insoles.								
□ Prescribing medicine.								
□ Doing X-rays.								
□ Treating foot wounds.								
□ Does not know		□ Does not answer						
NOTE: If you wish to make an observation about any of the items proposed or to propose a new one, you can do so here:								

“Your help is essential to achieve the aim of this study, which is why we thank you enormously for your collaboration”.

## Appendix C. Validated Questionnaire

EDUCATION FOR PODIATRIC HEALTH IN PATIENTS WITH RHEUMATOID ARTHRITIS Nº QUESTIONNAIRE:
The data obtained in this study will be confidential and will be dealt with in accordance with the provisions of the current legislation. Many thanks for your collaboration.

Age: _____	Gender Male Female	Marital status Single Divorced Married Widow/widower Other: ______	Years being treated for Rheumatoid Arthritis: _____
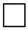

**Please mark your answer with an X. MARK AN OPTION.**
**1. Does Rheumatoid Arthritis produce pain in feet as well as in other parts of the body?**
Yes No Does not know Does not answer
**2. Does Rheumatoid Arthritis produce deformities in feet such as: claw toes, bunions, corns, etc.?**
Yes No Does not know Does not answer
**3. Rheumatoid Arthritis can produce falls and cause difficulty in walking etc.?**
Yes No Does not know Does not answer
**4. Would it be beneficial for the health of your feet to regularly use quality standard sports shoes?**
Yes No Does not know Does not answer
**5. Would daily use of wide footwear, with a soft sole, with a fastening system (laces, Velcro, zips, etc.) and a low heel be beneficial?**
Yes No Does not know Does not answer
**6. Should you apply moisturizing cream daily?**
Yes No Does not know Does not answer
**7. Is it appropriate to dry between your toes after your daily bath?**
Yes No Does not know Does not answer
**8. What is the best way of cutting your toenails?**
Straight, trimming the tips. Curved, trimming the tips.
Straight, without trimming the tips. Does not know Does not answer
**9. Is it appropriate to treat calluses and corns yourself?**
Yes No Does not know Does not answer
**10. Could the intervention of a podiatrist improve your general wellbeing by acting on your feet?**
Yes No Does not know Does not answer
**11. What’s the podiatrist’s role?**
**Please mark your answer with an X. MARK ONE OR VARIOUS OPTIONS.** Cutting nails. Getting rid of calluses and corns. Foot surgery. Studying footsteps. Making insoles. Prescribing medicine. Doing X-rays. Healing foot wounds. Does not know. Does not answer.
**12. What do you do when your feet hurt you? PLEASE WRITE YOUR ANSWER.**

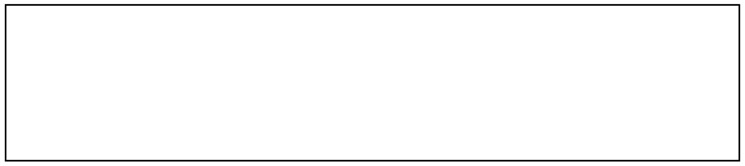

MANY THANKS FOR YOUR COLLABORATION
